# Senescence and impaired DNA damage responses in alpha-synucleinopathy models

**DOI:** 10.1038/s12276-022-00727-x

**Published:** 2022-02-08

**Authors:** Ye-Seul Yoon, Jueng Soo You, Tae-Kyung Kim, Woo Jung Ahn, Myoung Jun Kim, Keun Hong Son, Diadem Ricarte, Darlene Ortiz, Seung-Jae Lee, He-Jin Lee

**Affiliations:** 1 Department of Anatomy, Konkuk University, Seoul, 05029 Korea; 2 Department of Biochemistry, Konkuk University, Seoul, 05029 Korea; 3 Research Institute of Medical Science, Seoul, 05029 Korea; 4 IBST, Konkuk University, Seoul, 05029 Korea; 5 Department of Biomedical Sciences and Neuroscience Research Institute, Seoul National University College of Medicine, Seoul, Korea; 6 Departments of Exercise Physiology and Biochemistry, Korea National Sport University, Seoul, Korea; 7 Department of Microbiology, College of Natural Sciences, Dankook University, Seoul, Korea

**Keywords:** Parkinson's disease, Neurodegeneration

## Abstract

α-Synuclein is a crucial element in the pathogenesis of Parkinson’s disease (PD) and related neurological diseases. Although numerous studies have presented potential mechanisms underlying its pathogenesis, the understanding of α-synuclein-mediated neurodegeneration remains far from complete. Here, we show that overexpression of α-synuclein leads to impaired DNA repair and cellular senescence. Transcriptome analysis showed that α-synuclein overexpression led to cellular senescence with activation of the p53 pathway and DNA damage responses (DDRs). Chromatin immunoprecipitation analyses using p53 and γH2AX, chromosomal markers of DNA damage, revealed that these proteins bind to promoters and regulate the expression of DDR and cellular senescence genes. Cellular marker analyses confirmed cellular senescence and the accumulation of DNA double-strand breaks. The non-homologous end joining (NHEJ) DNA repair pathway was activated in α-synuclein-overexpressing cells. However, the expression of MRE11, a key component of the DSB repair system, was reduced, suggesting that the repair pathway induction was incomplete. Neuropathological examination of α-synuclein transgenic mice showed increased levels of phospho-α-synuclein and DNA double-strand breaks, as well as markers of cellular senescence, at an early, presymptomatic stage. These results suggest that the accumulation of DNA double-strand breaks (DSBs) and cellular senescence are intermediaries of α-synuclein-induced pathogenesis in PD.

## Neurodegeneration: Parkinson’s protein impairs DNA repair capacity

Excess levels of a protein involved in Parkinson’s disease can impair the brain’s capacity to repair DNA damage, leading to a state of cellular aging that accelerates neuronal death. When aggregated, the α-synuclein protein plays a major role in Parkinson’s disease and other neurodegenerative disorders. A team from South Korea, led by He-Jin Lee of Konkuk University, Seoul, and Seung-Jae Lee of Seoul National University College of Medicine, showed that human neuronal cells and mouse models with elevated expression of α-synuclein develop double-stranded breaks in their genomes as a consequence of deficient quality control mechanisms. The accumulated DNA damage spurs the cells to enter a state in which they show canonical signs of cellular aging but remain metabolically active in ways that fuel neurodegeneration. Therapies that target these processes could help prevent or treat α-synuclein–linked diseases.

## Introduction

α-Synuclein is an abundant neuronal protein with an intrinsically disordered structure^1^. This protein is abnormally folded and aggregated in several neurodegenerative diseases, referred to as α-synucleinopathies, such as dementia with Lewy bodies, multiple system atrophy, and Parkinson’s disease (PD)^2^. These diseases affect millions of people worldwide, with the distinct characteristics of intracytoplasmic protein aggregates and a gradual increase in neuronal death in particular areas of the brain.

Aging is a serious risk factor for PD and related neurodegenerative diseases^3^. The central nervous system undergoes considerable changes with age. Brain autopsy studies of aged people without a PD diagnosis have reported brain and spinal cord atrophy; decreases in the volume of gray matter; accumulation of pathological protein aggregates, such as amyloid plaques, neurofibrillary tangles, and Lewy bodies; and inclusions of TAR DNA-binding protein 43 and senescent cells^4,5^.

Cellular senescence is defined by irreversible cell cycle arrest and resistance to apoptotic death, often accompanied by changes in cell metabolism, such as changes in protein synthesis, glycolysis, fatty acid oxidation, reactive oxygen species (ROS) generation, and the acquisition of senescence-specific phenotypes^3^. In addition, senescent cells undergo morphological alterations to become larger and irregular due to cytoskeletal rearrangements and changes in cell membrane composition^6,7^. Senescence is often triggered by irreparable DNA damage, which accumulates with aging. This accumulation may be responsible for the pathogenesis of age-related diseases, such as neurodegenerative diseases.

DNA double-strand breaks (DSBs) arise infrequently, on the order of 10–50 per cell per day^8^. However, they are highly toxic and mutagenic, as chromosomal breakage may result in loss of genetic integrity^9^. Two major pathways are involved in repairing DSBs: homologous recombination (HR) and non-homologous end joining (NHEJ). HR is the predominant DSB repair pathway during embryonic development, meiotic recombination, replication fork stabilization, one-ended DSB repair, and two-ended DSB repair of the late S/G2 phase of the cell cycle^10–12^. The NHEJ pathway is the main pathway in mammalian cells acting on DSBs through all cell cycle phases, including the G1 interphase^12^. The primary DSB repair pathway for postmitotic neurons is NHEJ, although HR is important for other proliferating cells in the brain^9,13–16^. The initiation of DSB repair involves the formation of the MRE11-RAD50-NBS1 (MRN) complex, a central DSB sensor^17,18^. MRE11 plays an important role in the regulation of the choice of DSB repair pathway^19^. DNA end resection of DSBs leads to the loading of the RPA complex and RAD51 for HR repair, whereas blocking resection leads to NHEJ repair^19^. The binding of the Ku70/Ku80 heterodimer to DSB sites is recognized as blocking resection, which leads to NHEJ repair.

DNA damage activates a series of cellular pathways called DNA damage responses (DDRs). DSBs are the strongest triggers for such reactions^20^. In addition to the direct restoration of DNA integrity, DDRs also activate several cellular processes, such as cell cycle checkpoints, gene expression, and protein turnover. p53/p21 and p16/p16INK4a-pRB are the two primary regulators of these responses. Poor execution of DDRs may trigger cellular senescence; persistent DDRs may lead to age-related neurodegenerative diseases.

This study aimed to assess the effects of α-synuclein on DDRs and their connections to cellular senescence. Our results showed that α-synuclein in human neuronal cells increased DSBs with impaired DNA repair. α-Synuclein-induced impairment of DSB repair leads to increased levels of senescence markers. These results suggest that α-synuclein induces cellular senescence with DSB accumulation via impaired DDRs.

## Materials and methods

### Materials

The following primary antibodies were used: α-synuclein monoclonal antibody (BD Biosciences), γH2AX, phospho-ATM, ATM, p53, H3, poly(ADP-ribose) polymerase (PARP), ERCC1, XRCC1, MRE11, Rad51, Ku80, DDB2, p16, p73, and H3K9me3 antibodies (Abcam, Cambridge, UK); H2AX, phospho-p53, p21, and Ku70 antibodies (Cell Signaling Technology, Danvers, MA, USA); α-tubulin and β-actin antibodies (Sigma–Aldrich Corp., St. Louis, MO, USA); and 53BP1 antibody (Novus Biologicals, Centennial, CO, USA).

Retinoic acid, poly-L-lysine, and glutathione were obtained from Sigma–Aldrich. NE-PER™ Nuclear and Cytoplasmic Extraction Reagents were obtained from Thermo Fisher Scientific (Waltham, MA, USA). X-gal was obtained from Duchefa Biochemie (Haarlem, Netherlands).

### RNA-seq analysis

#### Transcriptome analysis

Total RNA was amplified and purified using the Target Amp-Nano Labeling Kit for Illumina Expression BeadChip. Detection of the array signal was carried out using Amersham Fluorolink Cy3 Streptavidin according to the bead array manual. The arrays were scanned using a bead array reader confocal scanner. The quality of the hybridization and overall chip performance were monitored manually by visual inspection of internal quality control checks and raw scanned data. Raw data were extracted using the software provided by Illumina Genome Studio v2011.1 and Gene Expression Module v1.9.0. Array probes were logarithm transformed and normalized using the quantile method. Filtered reads were aligned to the human reference genome (hg38 assembly) using the STAR mapper. The mapped reads were counted and converted to TPM values using RSEM. For differentially expressed gene analysis, fold-change and statistical significance were calculated using DESeq. Gene set enrichment analysis (GSEA) was performed in preranked mode. This dataset was obtained from the National Center for Biotechnology Information (NCBI) database (accession no. GSE149559).

#### Gene Ontology analysis

DAVID, Metascape, and Enrichr were used to infer the biological functions of the genes associated with the peaks. Default parameters were used.

#### Rank–rank hypergeometric overlap (RRHO)

To evaluate global gene expression profiles, the RRHO test was performed on two sets of gene expression comparisons (10.3390/ijms20236098). In this algorithm, genes were ranked according to their differential expression between two sample groups, and then these ranked gene expression profiles were iteratively assessed for overlap.

### Chromatin immunoprecipitation (ChIP) assay

ChIP assays were performed according to the instructions provided by Upstate Biotechnology. For each assay, 50 μg of DNA was sheared by sonication (DNA fragment size 200–500 bp) and precleared with protein A magnetic beads (Upstate Biotechnology #16-661). Then, 50 μg of DNA was precipitated using γH2AX and P53 antibodies. After immunoprecipitation (IP), the recovered chromatin fragments were subjected to sequencing.

#### Library preparation and sequencing

The library was constructed using the NEBNext® Ultra^TM^ DNA Library Prep Kit. Briefly, the chipped DNA was ligated to adaptors. After purification, PCR was performed on the adaptor-ligated DNA with an index primer for multiplex sequencing. The library was purified using magnetic beads to remove all the reaction components. The size of the library was assessed using an Agilent 2100 Bioanalyzer. High-throughput 100 bp paired-end sequencing was performed using a HiSeq 2500 system. The dataset was submitted to the NCBI Gene Expression Omnibus database (accession no. GSE149558).

#### ChIP-seq data analysis

The sequenced reads were trimmed using BBMap (BBDuk) and aligned to the human reference genome (hg38 assembly) using Bowtie 2. HOMER (findPeaks) was used to identify P53 binding sites (peaks) or rH2AX-enriched sites compared with the corresponding input samples in Con and SNCA-overexpressing cells with a false discovery rate-adjusted cutoff value of 0.001. The identified peaks were annotated using a known gene database (RefSeq). The annotated peaks were categorized into two groups (promoters and enhancers). Peaks located between −1 and +0.1 kb from the transcription start site were defined as promoter peaks, while the remaining peaks were defined as enhancer peaks. Superenhancer regions were also identified using HOMER (findPeaks with “super” option). The read coverage tracks for visualization were constructed using HOMER (UCSC file) with default options.

#### Motif analysis

Motif analysis of γH2AX-bound sequences depending on genomic locations (promoter and enhancer) was performed using HOMER (findMotifsGenome.pl) with the default option.

### Animal studies

The human alpha-synuclein (A53T) transgenic line G2-3 was described previously^21^. B6.Cg-tg (Prnp-SNCA*A53T)23Mkle/J hemizygous mice overexpress mutant α-synuclein in the brain at levels approximately sixfold higher than the level of endogenous mouse α-synuclein. Three- and 8.5-month-old mice expressing A53T and control C57BL/6J mice were purchased from Jackson Laboratory (Bar Harbor, ME, USA). All mice were housed in pathogen-free facilities under 12-h light/12-h dark cycles with ad libitum access to food and water. All experimental animals were handled in accordance with the animal care guidelines of Konkuk University (IACUC KU16067-2).

### Cell culture

The human neuroblastoma cell line SH-SY5Y (ATCC CRL-2266) was maintained and differentiated as described previously^22^.

### α-Synuclein, green fluorescent protein (GFP), and LacZ expression

Differentiated SH-SY5Y cells were transduced with recombinant adenoviral vectors (adeno/α-syn [m.o.i. 33–50, depending on the batch], adeno/lacZ, or adeno/GFP), as previously described^22,23^.

### Preparation of cell extracts

Cell extracts were obtained as described previously^24^. Briefly, cells were rinsed with ice-cold phosphate-buffered saline (PBS), and ice-cold extraction buffer (PBS/1% Triton X-100/protease inhibitor cocktail/phosphatase inhibitor cocktail) was added. After incubating on ice for 10 min, the cell extracts were centrifuged at 16,000 × *g* for 10 min, and the supernatants and pellets were reserved separately for further analysis.

### Isolation of nuclear and cytoplasmic extracts

Nuclear extracts were prepared using the NE-PER Nuclear Cytoplasmic Extraction Reagent Kit according to the manufacturer’s instructions. Briefly, cells were rinsed with ice-cold PBS, and cytoplasmic extraction buffer was added. Cells were collected using a cell scraper. After incubating on ice for 10 min, the cell extract was centrifuged at 16,000 × *g* for 10 min. The supernatant fraction (cytoplasmic extract) was transferred to a prechilled tube. The insoluble pellet fraction was resuspended in nuclear extraction reagent and vortexed vigorously. The nuclear and cytoplasmic extracts were stored at −80 °C until further analysis.

### Western blotting

Western blotting was performed as described previously^24^. Chemiluminescence detection was performed using a FUJIFILM Luminescent Image Analyzer LAS-3000 and GE Healthcare Amersham Imager 680. Images were analyzed using the Multi Gauge (v3.0) software (Fujifilm, Tokyo, Japan).

### Cell viability assay

The cells were trypsinized and divided into two tubes. Accustain solution T containing detergent and propidium iodide (PI) was added to one tube to determine the total number of cells. Accustain solution N, containing PI but no detergent, was added to the other tube to label the damaged cells. The cells from both tubes were counted using an ADAM cell counter (NanoEnTek, Seoul, Korea).

### Total RNA extraction and quantitative real-time PCR

Total RNA was extracted using the RNeasy Mini Kit (Qiagen, Hilden, Germany). The RNA extract was reverse transcribed using the High Capacity cDNA Reverse Transcription Kit (Applied Biosystems, Foster City, CA, USA). Quantitative real-time PCR was performed on a LightCycler 480 II using LightCycler 480 SYBR Green I Master Mix (Roche, Basel, Switzerland), as recommended.

The primers used were as follows: DDB2 forward: 5′-AAACCCAGAAGACCTCCGAG-3′, DDB2 reverse: 5′-ACATCTTCTGCTAGGACCGG-3′, BTG2 forward: 5′-AGGGAACCGACATGCTCC-3′, BTG2 reverse: 5′-GGGAAACCAGTGGTGTTTGT-3′, RPS27L forward: 5′-ACTACATCCGTCCTTGGAAGAG-3′, RPS27L reverse: 5′-GCTGAAAACCGTGGTGATCT-3′, p21 forward: 5′-CACCGAGACACCACTGGAGG-3′, p21 reverse: 5′-GAGAAGATCAGCCGGCGTTT-3′, GAPDH forward: 5′-GAGTCAACGGATTTGGTCGT-3′, GAPDH reverse: 5′-TGGAAGATGGTGATGGGATT-3′, p21_1 forward: 5′-CACCGAGACACCACTGGAGG-3′, p21_1 reverse: 5′-GAGAAGATCAGCCGGCGTTT-3′, GAPDH forward: 5′-GAGTCAACGGATTTGGTCGT-3′, and GAPDH reverse: 5′-TGGAAGATGGTGATGGGATT-3′.

(tissue extract) p16 forward: 5′-TTCTTGGTGAAGTTCGTGCG-3′, p16 reverse: 5′-GCACCGTAGTTGAGCAGAAG-3′, p21_2 forward: 5′-ACAAGAGGCCCAGTACTTCC-3′, p21_2 reverse: 5′-GTTTTCGGCCCTGAGATGTT-3′, p53 forward: 5′-TGCTCACCCTGGCTAAAGTT-3′, and p53 reverse: 5′-AATGTCTCCTGGCTCAGAGG-3′.

### Senescence-associated beta-galactosidase staining

The cells were rinsed with ice-cold PBS and fixed in 4% paraformaldehyde (PFA) in PBS. The cells or tissues were incubated overnight at 37 °C (without CO_2_) in a freshly prepared X-gal staining solution. The stained samples were rinsed with ice-cold methanol, air-dried, and then imaged using a digital camera.

### Immunofluorescence cell staining

The cell staining procedure was described previously^24^. Briefly, cells grown on poly L-lysine-coated coverslips were fixed in 4% PFA in PBS. The fixed cells were permeabilized with 0.1% Triton X-100 and then incubated in blocking solution (5% bovine serum albumin and 3% goat serum in PBS). Primary antibodies were diluted in blocking solution and added to the cells. After washing in PBS, the cells were incubated with Alexa488, Cy2, rhodamine red X, or Alexa647 fluorescent dye-conjugated secondary antibodies (Jackson ImmunoResearch Laboratories, PA, USA) and then washed again in PBS. The nuclei were stained with TO-PRO-3 dye (Thermo Fisher Scientific, MA, USA) or Hoechst 33342 (Sigma) and then mounted under coverslips using ProLong^TM^ Gold Antifade reagent (Thermo Fisher Scientific). The stained cells were observed under an Olympus FV1000 confocal laser-scanning microscope or Zeiss LSM 900 with Airyscan 2.

### Immunohistochemistry of brain tissues

Mouse brain tissue was kept in 4% PFA in cold 0.1 M phosphate buffer (pH 7.4) for 2 days, followed by incubation in 30% sucrose solution. For immunostaining, 40-μm coronal sections were cut on a sliding microvibratome (Leica, Germany). The details of the immunohistochemistry procedures have been described elsewhere^25^. Briefly, 40-µm-thick floating brain sections were quenched with 0.3% H_2_O_2_ and then blocked with 4% BSA in PBST (0.1% Triton X-100). The samples were incubated overnight at 4 °C with primary antibodies against mouse anti-phospho-α-synuclein (pS129; BioLegend, CA, USA, #825701, 1:500) and rabbit anti-γH2AX (Abcam, MA, USA, #ab111741, 1:500). After washing with PBST, the brain sections were incubated with biotinylated secondary antibodies (Bio–Rad, #170-6515, #170-6516, 1:200) and treated with avidin–biotin peroxidase complex (ABC Elite kit, Vector Laboratories, CA, USA). Then, 3,3-diaminobenzidine (DAB)-developed sections were observed under a ZEISS AX10 microscope. All samples were evaluated by optical density analysis using the ImageJ program (NIH) with correction for background signal levels.

### Immunofluorescence of brain tissues

Details of the immunohistochemistry procedures are provided elsewhere^26^. Briefly, 40-µm-thick floating brain sections were quenched with 0.3% H_2_O_2_ and then blocked with 4% BSA in PBST (0.1% Triton X-100). Samples were incubated overnight at 4 °C with primary antibodies against mouse anti-phospho-α-synuclein (pS129; BioLegend, CA, USA, #825701, 1:500), rabbit anti-γH2AX (Abcam, MA, USA, #ab111741, 1:200), and mouse anti-NeuN (Merck Millipore, Germany, #MAB377, 1:500). After washing in PBST, the brain sections were incubated with Alexa488- or rhodamine red-X-conjugated secondary antibodies (Jackson ImmunoResearch Laboratories, PA, USA) and then washed again with PBST.

Stained sections were mounted in a fluorescence mounting medium containing DAPI (Vector Laboratories, CA, USA). The stained samples were observed under a Carl Zeiss LSM 700 confocal laser-scanning microscope.

### Electron microscopy

SH-SY5Y cells were transduced with adeno/α-syn or adeno/lacZ for 3 days. The sections were prepared as described previously^27^.

### Statistical analysis

All experiments were repeated at least three times. The values were expressed as the mean ± S.E.M. Null hypotheses of no difference were rejected if the *P* values were <0.05. The data were analyzed using repeated-measures one-way analysis of variance with posttests using the Prism 9 software (GraphPad Software Inc., CA, USA). *P < 0.05, **P < 0.01, ***P < 0.001, ****P < 0.0001).

## Results

### Gene expression analysis of SH-SY5Y human neuroblastoma cells with ectopic α-synuclein expression

**Fig. 1:**
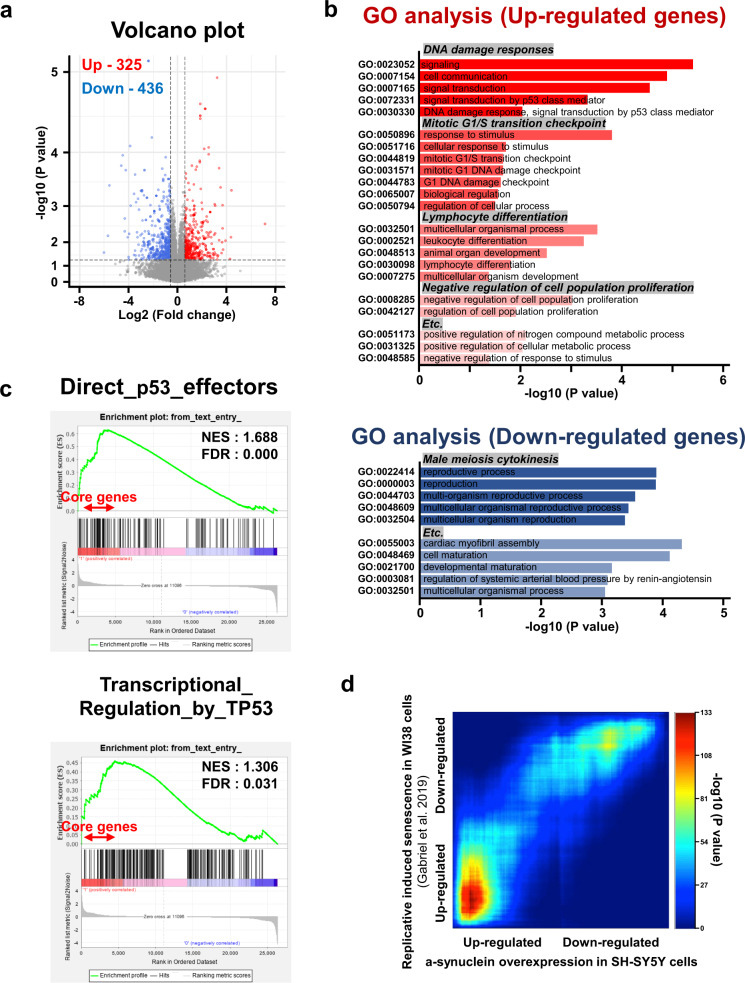
Transcriptome analysis of ectopic α-synuclein expression in SH-SY5Y neuroblastoma cells. **a** Volcano plot for differentially expressed genes (DEGs): fold change ≥1.5, *P* < 0.05. **b** Gene Ontology (GO) analysis of DEGs. **c** Gene set enrichment analysis of α-synuclein overexpression in SH-SY5Y cells. **d** Heatmaps of enrichment rank–rank hypergeometric overlap analysis with senescent cells.

To investigate the α-synuclein-dependent transcriptional landscape, RNA sequencing was performed after the expression of α-synuclein in SH-SY5Y cells. Over 325 genes were upregulated and 436 genes were downregulated upon α-synuclein expression (Fig. 1a, red and blue colored dots, fold change ≥1.5, *P* < 0.05). Downregulated genes were enriched in meiosis cytokinesis of unknown function (Fig. 1b). In contrast, the upregulated genes were highly involved in DDRs, the mitotic G1/S transition checkpoint, lymphocyte differentiation, and negative regulation of cell proliferation (Fig. 1b). GSEA of α-synuclein expression showed that the gene expression signature was positively associated with direct TP53 effectors and transcriptional regulation by TP53, although the normalized enrichment score value was not significant (Fig. 1c). We further validated the transcriptional expression changes of DDB2, BTG2, and RPS27L, which belong to the core DNA repair gene set, by performing real-time reverse transcriptase–PCR after α-synuclein induction (Supplementary Fig. 1a).

Given that α-synuclein expression produced a senescence-like transcriptome signature, we performed RRHO analysis with induced senescent WI38 cells^28,29^ (Fig. 1d). The results showed a strong similarity between α-synuclein-induced and senescence genes, especially in the upregulated gene category. We confirmed that RRHO common genes belonged to the DNA repair, cell cycle, and transcription categories by performing a Reactome analysis (Supplementary Fig. 1b). These data suggest that α-synuclein expression may play a vital role in the nucleus via transcriptional regulation of genes involved in senescence and DDRs.

### Transcriptional regulation of γH2AX and p53 by promoter binding

**Fig. 2:**
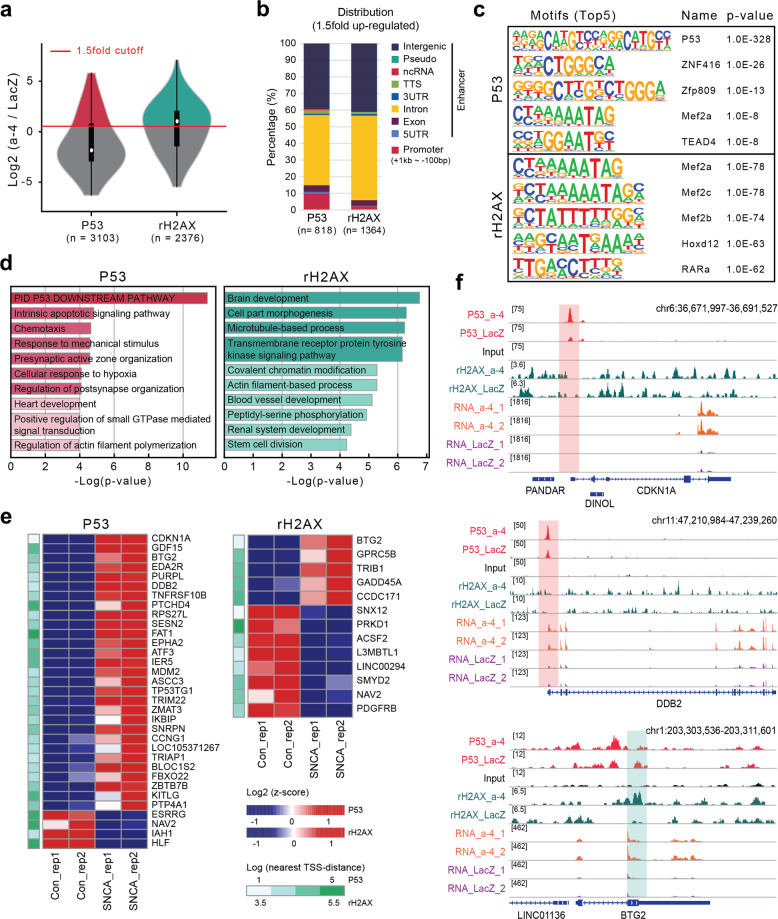
P53 and γH2AX ChIP-seq analysis combined with transcriptome data. **a** Violin plot of P53 and γH2AX enrichment upon SCNA overexpression. **b** Genomic distribution of increased binding of P53 and γH2AX upon SCNA overexpression (promoter −1 kb to +100 bp). **c** Motif analysis of enhanced P53 and γH2AX binding upon SCNA overexpression. **d** GO analysis of enhanced P53 and γH2AX binding upon SCNA overexpression. **e** Heatmap showing a list of DEGs with increased binding of P53 and γH2AX. The green bar indicates the distance between P53 and γH2AX peaks, with gene transcriptional start sites indicated. **f** Representative screenshot of the genes common to differentially expressed genes and increased binding of P53 or γH2AX upon SNCA overexpression.

To further delineate the transcriptional effects of α-synuclein, ChIP-seq of p53 and γH2AX was performed. Both proteins are well-known mediators of senescence and DDR. Line plots and binding heatmaps showed that p53 and γH2AX mapped well to the chromosomal regions (Supplementary Fig. 2). We focused on the regions showing a more than 1.5-fold increase in p53 and γH2AX peaks upon α-synuclein expression (Fig. 2a). The genomic distributions of p53 and γH2AX are shown in Fig. 2b. Increased p53 binding was enriched in promoter regions compared with γH2AX. Motif analysis validated the results, showing that a known p53 binding motif was one of the top p53 ChIP-seq motifs, followed by other zinc finger domain protein recognition sites (Fig. 2c). Notably, the Mef family occupied the top positions of the γH2AX enrichment motifs (Fig. 2c). Functional annotation of genes with increased p53 binding showed distinctive processes, such as the p53 downstream pathway, apoptotic signaling pathway, and chemotaxis (Fig. 2d). Increased levels of γH2AX were observed at genes associated with brain development and cell morphogenesis (Fig. 2d). These results suggest that elevated levels of α-synuclein may have toxic effects.

Next, we compared the differentially expressed genes whose promoters simultaneously exhibited increases in p53 or γH2AX binding to identify more significant α-synuclein transcriptional targets. We found that increased binding of p53 to the promoters tended to upregulate the expression further, including for the CDKN1A gene (29 genes upregulated and 4 genes downregulated). In contrast, γH2AX-enriched genes were slightly more often downregulated (five upregulated genes and eight downregulated genes) (Fig. 2e). A representative screenshot of CDKN1A, DDB2, and BTG2 in our RNA-seq and ChIP-seq data is shown in Fig. 2f. Taken together, the integrated analysis of P53 and γH2AX ChIP-seq combined with RNA-seq demonstrated that increased α-synuclein expression might trigger cellular senescence and the activation of the DDR pathway via p53.

### Changes in p53 and p21 levels upon α-synuclein expression

**Fig. 3:**
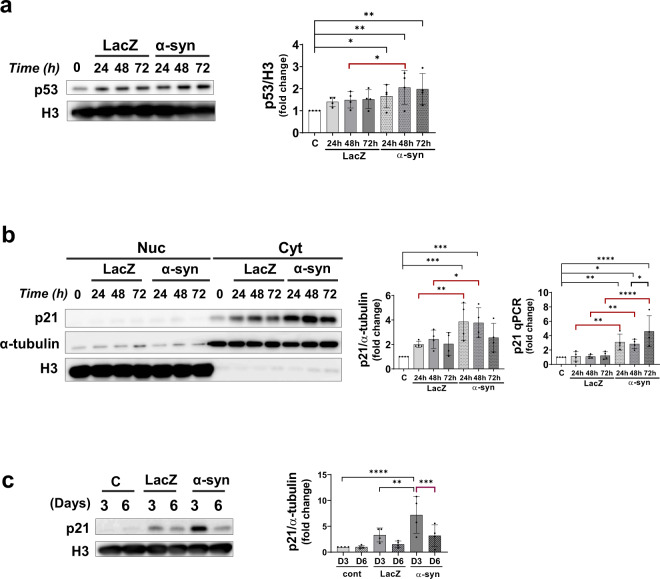
α-Synuclein expression upregulates p53 and p21 proteins. **a** p53 protein levels increased with α-synuclein expression in a time-dependent manner. **b** p21 mRNA and protein levels were elevated with α-synuclein protein overexpression. **c** p21 protein levels increased on Day 3 but were reduced to control (lacZ) levels on Day 6.

To verify whether p53 expression increased in α-synuclein-expressing cells, recombinant α-synuclein viral vector (adeno/α-syn) or control vector (adeno/lacZ) was transduced into differentiated SH-SY5Y cells. As shown in Fig. 3a, the p53 protein levels were significantly increased in cells expressing α-synuclein. Likewise, the levels of the p21 protein, which is downstream of p53, were highly elevated in the cytoplasm of α-synuclein-expressing cells, as shown in Fig. 3b. The p53 and p21 proteins peaked at 48 h after α-synuclein expression. The levels of p21 increased significantly on Day 3 but decreased considerably after Day 6 (Fig. 3c). This result is consistent with that of previous studies, which reported that p21 and p53 activation in senescent cells was transient and that their levels decreased after the establishment of growth arrest^30,31^.

### Premature senescence induced by α-synuclein expression

**Fig. 4:**
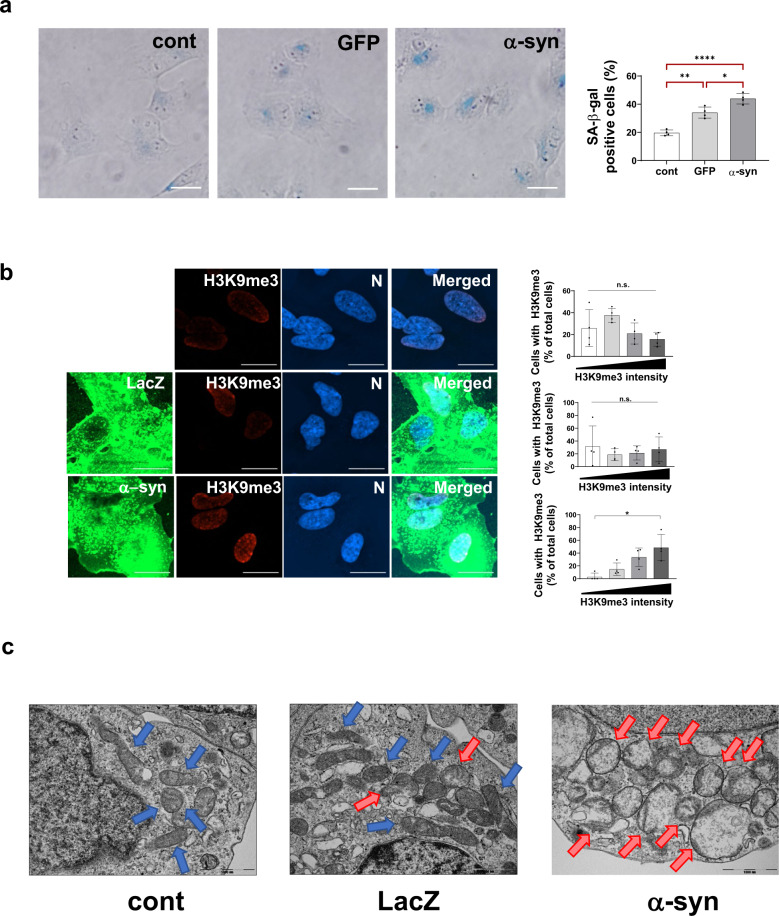
α-Synuclein-induced premature cellular senescence. **a** SA-b-gal assay showing increased staining (blue) in α-synuclein-expressing SH-SY5Y cells. **b** H3K9me3 (red) staining showing elevated heterochromatin DNA foci in α-synuclein-expressing cells (scale bar: 20 μm). The measured H3K9me3 intensity (intensity units; i.u.) was categorized as described (white bar: 0–2000 i.u.; light gray bar: 2001–8000 i.u.; dark gray bar: 8001–15500 i.u.; black bar: >15,501 i.u.). **c** TEM images showing mitochondria morphologies. Note that normal-appearing mitochondria are indicated by blue arrows, whereas abnormal mitochondria are indicated by red arrows.

The p53/p21 pathway is involved in several cellular activities that lead to cell death or survival. These include apoptosis, cell cycle arrest, repair, and senescence. Our results in Figs. 1 and 2 show that cellular senescence genes are upregulated at the transcriptional level. To determine whether α-synuclein overexpression induces cellular senescence, we performed a series of senescence detection assays. Senescence-associated β-galactosidase (SA-β-gal) is an inducible lysosomal enzyme that is produced at high levels during senescence. α-Synuclein expression in SH-SY5Y cells for 72 h increased the SA-β-gal levels by ~44% compared with the control (no overexpression; ~20%) and GFP expression (~34%) (Fig. 4a).

Another characteristic of cellular senescence is an increase in the number of the transcriptionally inactive senescence-associated heterochromatic foci, which are marked by histone H3 trimethylated at lysine 9 (H3K9me3)^32^. SH-SY5Y cells were transduced with adeno/α-syn and adeno/lacZ for 3 days and stained for H3K9me3 (Fig. 4b). The control (without transduction) and lacZ-expressing cells did not show an increase in H3K9me3 staining. In contrast, α-synuclein-expressing cells showed a gradual increase in the number of cells with enhanced H3K9me3 staining.

Often, senescent cells display enlarged mitochondria with misshapen cristae that are forced to the mitochondrial periphery^33^. Transmission electron microscopy results showed that the number of abnormal mitochondria was increased in α-synuclein-expressing cells (red arrows). The control and lacZ-expressing cells with normal mitochondria are indicated by blue arrows (Fig. 4c).

### DNA damage is induced by α-synuclein expression

**Fig. 5:**
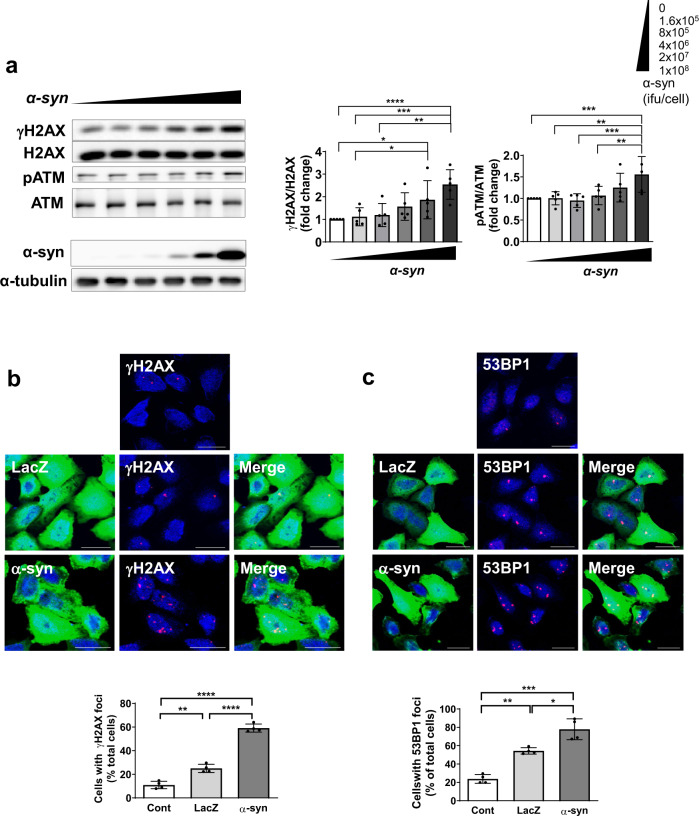
Increased DSBs correlated with increased α-synuclein expression levels. **a** γH2AX and phosphorylated ATM (pATM), DSB-binding proteins, increased with α-synuclein expression in a dose-dependent manner. Cells showed increased γH2AX (**b**) and 53BP1 (**c**) foci in α-synuclein-expressing cells (scale bar: 20 μm).

DNA DSBs are a powerful trigger for DDR. Depending on the severity of the damage, DDRs can lead to DNA repair and recovery, cellular senescence, or apoptosis^34^. As cellular senescence was observed upon α-synuclein expression, the extent of DNA damage was examined by measuring DSBs, which can be monitored based on the levels of γH2AX (Fig. 5a). α-Synuclein expression was regulated by lower to higher doses of recombinant adenoviral vector, as shown in Fig. 5a (lower panel). The γH2AX levels increased as α-synuclein levels increased. We also examined the levels of another DSB marker, the phosphorylated form of ataxia telangiectasia mutated (pATM). The ATM gene encodes a multifunctional phosphatidylinositol 3-kinase-like protein kinase, which is recruited to DSB sites as an inactive dimer. Upon autophosphorylation, the ATM dimer dissociates into kinase-active monomers to promote DNA repair^35,36^. Our results showed that ATM phosphorylation increased in a pattern similar to that of γH2AX, although to a lesser extent (~1.5-fold in pATM compared with the 2.5-fold in γH2AX) (Fig. 5a).

Another measure of DSBs is the formation of nuclear foci containing γH2AX. As shown in Fig. 5b, the γH2AX foci were more extensive and abundant in α-synuclein-expressing cells (~60%) than in LacZ (~25%) and control (~10%) cells. P53-binding protein 1 (53BP1) also forms large foci near DNA lesions^37^. The number of 53BP1 nuclear foci was also significantly increased in α-synuclein-expressing cells (Fig. 5c). These results suggest that an increase in α-synuclein expression leads to the accumulation of DNA DSB damage.

Confirming the increased level of DNA damage, PARP1, an upstream marker of both single-strand breaks (SSBs) and DSBs, was upregulated 24 h after α-synuclein expression but slowly decreased thereafter (Supplementary Fig. 3a, b). Of the two DSB repair pathways, HR occurs in cells that undergo the cell cycle for proliferation, whereas NHEJ is the primary DSB repair mechanism for postmitotic cells, such as neurons. The levels of the HR-related protein Rad51 seemed to be decreased, rather than increased, upon α-synuclein expression, ruling out the possibility of HR pathway activation (Supplementary Fig. 3c). We also examined whether SSB repair pathways were affected by α-synuclein expression (Supplementary Fig. 3d, e). The two SSB markers evaluated, ERCC1 (involved in nucleotide excision repair) and XRCC1 (involved in base excision repair), did not significantly change after α-synuclein expression. These results suggest that elevated α-synuclein levels specifically affect the NHEJ pathway more than other DNA damage repair pathways.

### α-Synuclein inhibits DNA repair genes

**Fig. 6:**
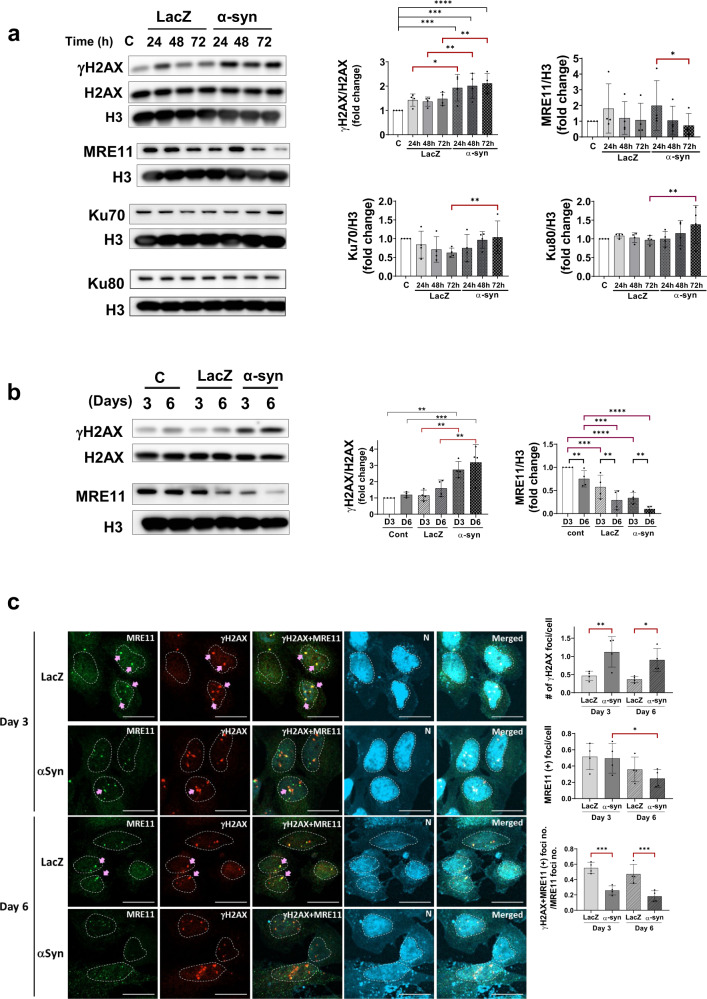
Reduced expression of DNA repair genes in α-synuclein-expressing SH-SY5Y cells. The elevation of the DSB marker protein γH2AX persisted throughout α-synuclein expression until Day 6 (**a**–**c**). In contrast, LacZ-expressing cells showed a transient increase in γH2AX that returned to normal after 48 h. The levels of the NHEJ repair pathway proteins Ku70 and Ku80 increased with α-synuclein expression and DSB accumulation (**a**). However, the level of the MRE11 protein, a critical component of the NHEJ repair pathway, decreased as α-synuclein levels were elevated (**a**–**c**) (scale bar: 20 μm).

Next, to determine whether DSBs could be effectively repaired, we examined DNA repair gene expression (Fig. 6). LacZ and α-synuclein induced the upregulation of γH2AX, a DSB marker, within 24 h (Fig. 6a). However, the increase in the levels of γH2AX in lacZ-expressing cells was transient, returning to baseline levels at later time points (48 and 72 h). In contrast, the γH2AX levels remained elevated for at least 6 days after α-synuclein expression (Fig. 6b). This result suggests that a temporary spike in DSBs could be caused by the expression of foreign proteins but that these were subsequently repaired, as shown with LacZ. However, DNA damage caused by α-synuclein may inhibit the repair process, and DSBs may persist and accumulate.

The Ku70 and Ku80 proteins are involved in the NHEJ DSB repair pathway. Our results (Fig. 6a) showed an increase in their expression in α-synuclein-expressing cells, suggesting that the NHEJ repair pathway is activated. To further assess the activation of the NHEJ pathway, we examined the expression levels of MRE11. MRE11 is a DSB repair nuclease that forms a complex with Rad 50 and Nbs1, that is, the MRN complex. The MRN complex acts as a sensor of DSBs, relocating from the cytosol to the DNA breakage site. The level of the MRN complex component MRE11 decreased significantly in the nuclear fraction with time after α-synuclein expression (Fig. 6a). The decrease in MRE11 expression continued for up to 6 days, as the levels of γH2AX remained increased (Fig. 6b). Immunofluorescence staining of γH2AX and MRE11 showed similar patterns (Fig. 6c). The number of γH2AX-positive foci increased significantly with α-synuclein expression and remained elevated until Day 6 (Fig. 6c, upper graph). Both the number of cells with γH2AX foci and the number of γH2AX foci per cell increased significantly even on Day 10 compared with control and lacZ-expressing cells (Supplementary Fig. 4). However, the number of MRE11-positive foci was reduced considerably on Day 6 relative to that on Day 3 (Fig. 6c, middle graph). The number of foci containing both γH2AX and MRE11 (pink arrows) also decreased significantly on Days 3 and 6 (lower graph, Fig. 6c), suggesting that several DNA DSB sites (γH2AX (+)) were missing the DNA repair proteins, leading to dysfunction of the DNA repair process. These results collectively suggest that α-synuclein overexpression causes DNA DSBs and that incomplete activation of the NHEJ repair pathway may lead to the accumulation of DNA damage.

### P53 and γH2AX expression increased in α-synuclein tg mice

**Fig. 7:**
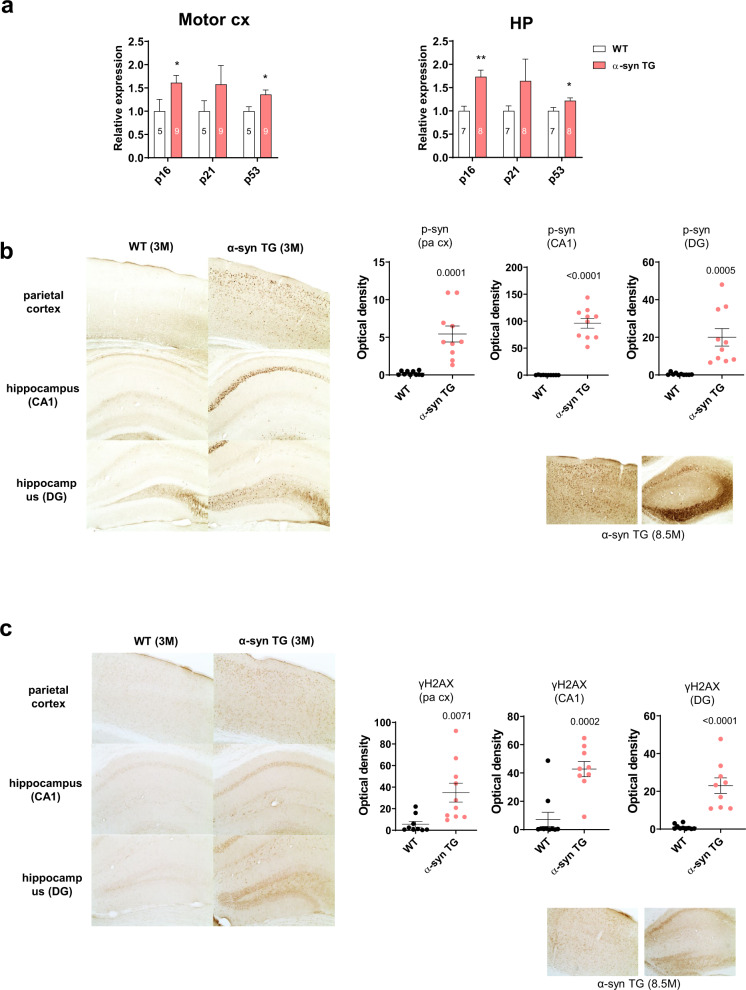
DSB changes in α-synuclein tg mice. **a** p16, p21, and p53 mRNA levels increased in the motor cortex (cx) and hippocampus (HP) in α-syn tg mice compared with those of wild-type mice. **b** Immunohistochemistry of WT and α-syn tg (3 months) mice with the anti-phospho-α-synuclein antibody. Note that phosphorylated α-synuclein increased in the parietal cortex, hippocampal CA1 region, and DG of α-synuclein tg mice. Phosphorylated α-synuclein increased more in the older tg (8.5 months) mice than in the 3-month tg mice. **c** Immunohistochemistry of WT and α-syn TG (3 months) mice with the anti-γH2AX antibody. Note that γH2AX increased in the parietal cortex, hippocampal CA1 region, and DG of α-synuclein tg mice. γH2AX increased more in the older tg (8.5 months) mice than in the 3-month tg mice.

**Fig. 8:**
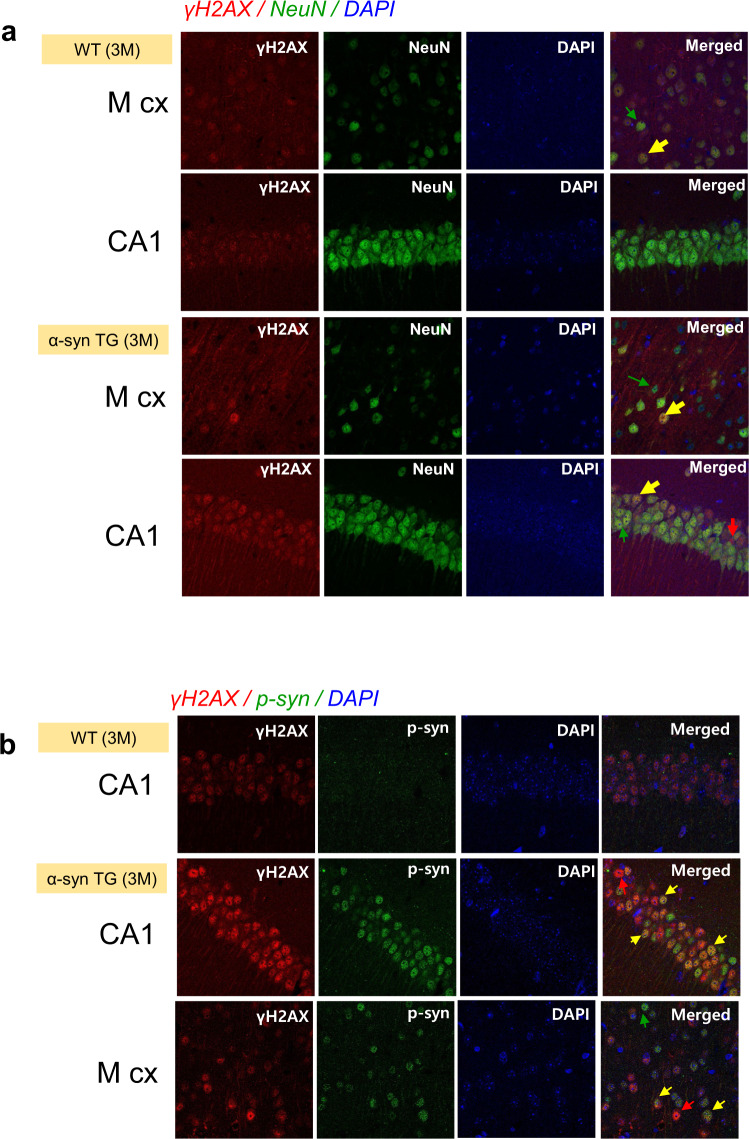
Localization of γH2AX and phosphorylated α-synuclein in the motor cortex and hippocampal regions in wild-type and α-synuclein tg mice. **a** Localization of γH2AX in neurons (NeuN positive). Most of the γH2AX was found in the neurons (yellow arrows), but some γH2AX-positive cells were not found in the neurons (red arrow). **b** Localization of γH2AX and phosphorylated α-synuclein. Most of the γH2AX was localized to cells with phosphorylated α-synuclein (yellow arrows), but some γH2AX-positive cells had no phosphorylated α-synuclein staining (red arrow).

In vivo, DNA damage by α-synuclein was observed in 3-month-old α-synuclein tg mice^21^ (Fig. 7). Total RNA was isolated from different areas of the brain for real-time PCR to test for the cellular senescence markers p16, p21, and p53 (Supplementary Fig. 5). The results showed that the expression of all three genes was elevated in the motor cortex (motor cx) and hippocampus (HP) of the tg mice compared with the wild-type mice (Fig. 7a). The parietal cortex and hippocampal CA1 and DG areas exhibited extensive staining of phosphorylated α-synuclein (p-syn) (Fig. 7b). Interestingly, these areas also presented a higher number of γH2AX foci-positive cells, suggesting that the DNA DSBs were elevated in these regions (Fig. 7c). Increased staining of phosphorylated α-synuclein and γH2AX was observed in older tg mice (8.5 months) than in the younger (3 months) tg mice (Fig. 7b, c; lower right panels). DNA damage is not restricted to neurons. NeuN-positive cells were stained for γH2AX (yellow arrows), whereas other γH2AX-positive cells were not stained with NeuN (red arrows) (Fig. 8a). The colocalization of γH2AX and p-syn was observed by conducting immunofluorescence staining in these areas (Fig. 8b). However, not all p-syn-positive cells were γH2AX-positive, nor were all γH2AX cells positive for p-syn. These results confirm the presence of DNA damage and senescence in the presymptomatic stages of α-synuclein tg mice.

## Discussion

The mechanism by which α-synuclein exerts its pathogenic actions in PD is of great importance for understanding the disease mechanism and for developing therapeutic strategies. Here, we showed that the overexpression of α-synuclein leads to cellular senescence and impaired DDRs in both in vitro and in vivo models of synucleinopathy. Transcriptome analysis using RNA sequencing revealed cellular senescence with activation of the p53 pathway and DDRs in α-synuclein-overexpressing cells. ChIP analyses confirmed that increased binding of p53 to the promoter regions upregulated the expression of cellular senescence- and DDR-related genes such as p21 (CDKN1A), DDB2, and BTG2. Cellular senescence and DNA DSBs were observed in cells overexpressing α-synuclein. Specifically, the NHEJ DNA repair pathway was partially activated, with a critical component of the pathway being reduced, resulting in a faulty DNA repair system. Histopathological analyses of α-synuclein transgenic mice showed increases in the levels of phospho-α-synuclein and DNA DSBs, as well as markers of cellular senescence at the presymptomatic stage, suggesting that cellular senescence and DNA damage are early events in synucleinopathy. The accumulation of DNA damage has been previously reported in α-synuclein-overexpressing cells and in several synucleinopathy mouse models^38–40^. DNA damage resulted in dopaminergic degeneration and motor deficits, similar to those observed in our study^38^. We built upon previous experiments, showing that α-synuclein expression induced DDRs. However, these responses are incomplete, leading to the accumulation of DNA damage and induction of cellular senescence.

The mechanism by which DNA damage causes cellular senescence remains unclear. However, accumulating evidence suggests that these two phenomena are strongly associated. DNA damage accumulates with aging in both senescent cells and aged mammalian tissues^41^. These cells and tissues retained unrepaired DNA DSBs that were associated with DDR markers. Therefore, persistent DNA damage is probably a consequence of incomplete DDRs. Consistent with this idea, the levels of the DNA repair proteins Ku70 and Mre11 were decreased in aging human lymphocytes^42^. Neurons with prolonged DDRs showed typical characteristics of cellular senescence, such as mitochondrial dysfunction, production of ROS, and metabolic abnormalities. Neurons are particularly vulnerable to DNA damage because they are postmitotic and are highly metabolically active. A large proportion of neurons in several brain areas of aged mice showed severe DNA damage. Interestingly, the senescent-like phenotypes of a mouse model of premature senescence were rescued by deleting the p21 gene, a key signal transducer in cellular senescence^43^. These results suggest that senescence-like phenotypes can be induced by severe DNA damage in mature neurons.

Prolonged or irreparable DNA damage leads to various human diseases that are characterized by genomic instability, including neurodegenerative diseases^20,44,45^. Impairment of the base excision repair (BER) pathway, especially of the mitochondrial BER pathway, can cause various neurodegenerative disorders, such as Alzheimer’s disease^9,46^. Ataxia-oculomotor apraxia-1 (AOA1) and spinocerebellar ataxia with axonal neuropathy-1 (SCAN1) are associated with SSB repair defects^9,47,48^. AOA1 is associated with mutations in a novel human gene, aprataxin^49,50^, which encodes nucleotide hydrolases/transferases^51^. The encoded protein may play a role in single-stranded DNA repair through its nucleotide-binding activity and its diadenosine polyphosphate hydrolase activity. A mutation in the DNA repair protein tyrosyl-DNA phosphodiesterase 1 (TDP1), which can repair abortive SSBs created by topo1, is associated with SCAN1^52^. Likewise, defects in DSB repair genes due to genetic mutations are also associated with neurological diseases, such as ataxia-telangiectasia (AT), AT-like disorder (MRE11 gene mutations), and Nijmegen breakage syndrome (NBS1 gene mutations)^9^. These genes are essential for the recognition of DSBs at the initial stage of DSB repair. These findings suggest that the human nervous system is vulnerable to DNA damage and that impairment of DNA repair pathways can cause neurological diseases.

Our study showed that the overexpression of α-synuclein leads cells to initiate DDRs. However, in this case, the DDRs are not fully functional, which leads to the accumulation of DNA damage, particularly DSBs. Lewy body-containing neurons in the brain tissues of human patients showed increased levels of DNA DSBs^53^. The precise mechanism by which α-synuclein overexpression causes defects in DDRs remains unknown. The levels of MRE11 were decreased in cells overexpressing α-synuclein. Mre11 is the nuclease component of MRN/X, one of the primary complexes responsible for recognizing and repairing DSBs as well as transducing DSB signals in eukaryotes. Elucidation of the pathway leading to the reduction of Mre11 would reveal the mechanism by which DNA damage accumulates during synucleinopathy.

Interestingly, Schaser et al. recently showed that the removal of α-synuclein in human cells and mice resulted in increased levels of DNA DSBs after bleomycin treatment and a reduced ability to repair DNA damage^53^. These authors also suggested that α-synuclein binds double-stranded DNA and facilitates NHEJ repair. Taken together, these findings indicate that both overexpression and deficiency of α-synuclein caused impaired DNA repair and accumulation of DNA damage. It remains unclear how these seemingly contradictory observations can be explained: either too much or too little α-synuclein leads to increased DNA damage. To maintain functional DNA repair systems, it may be essential to maintain α-synuclein levels within a specific range.

In our study, DNA DSBs accumulated in both phospho-α-syn-positive cells and phospho-α-syn-negative cells in α-synuclein tg mice. Moreover, DNA DSBs have been found in nonneuronal cells and neurons. These observations may indicate the occurrence of senescence-induced senescence, in which senescent phenotypes spread to neighboring cells^20,54^. Alternatively, these results suggest that DNA damage may not solely be the result of the cell-autonomous actions of α-synuclein but may also be induced by a non-cell-autonomous mechanism. α-Synuclein and its aggregate form can be secreted by neurons^55^. These secreted forms of α-synuclein are the culprit of the non-cell-autonomous actions of this protein, affecting neighboring neurons and glia^56,57^. Neurons that were exposed to extracellular α-synuclein showed signs of apoptosis^58^, while glial cells treated with α-synuclein showed inflammatory responses^56,57^. The latter is particularly interesting because DNA damage can induce inflammatory responses in innate immune cells^59,60^. Our study paves the way for further studies on the mechanism of non-cell-autonomous neuronal degeneration and glial inflammation triggered by extracellular α-synuclein.

In conclusion, we propose that the accumulation of DNA damage and cellular senescence may be key components in the pathogenesis of PD and other α-synuclein-related neurological diseases. DNA damage appears to accumulate due to incomplete and hence impaired activation of DNA repair pathways. How α-synuclein impairs the DNA repair system is a critical question arising from the current study. Other important questions include how DNA damage leads to cellular senescence in neurons and what the consequences of senescence processes are in terms of neuronal function and viability. Pursuing these questions could ultimately lead to an understanding of the pathogenic mechanism of synucleinopathies.

## Supplementary information

Supplementary Figures
