# Supplementary material for: Senescence and impaired DNA damage responses in alpha-synucleinopathy models
Source: Exp Mol Med. 2022 Feb 8;54(2):115–28. doi: 10.1038/s12276-022-00727-x (PMC8894476; doi:10.1038/s12276-022-00727-x)
Supplement: Supplementary file 1 — Supplementary Figures [file 12276_2022_727_MOESM1_ESM.pdf]

Supplementary Figure legends

Supplementary Figure 1. Additional Transcriptome analysis upon ectopic α-synuclein expression in SH-SY5Y neuroblastoma cells. (a) Validation of the differential expressed genes (DEGs) of DNA repair by qPCR. (b) Reactome analyses of common genes in the Rank-Rank Hypergeometric Overlap analysis.

Supplementary Figure 2. Genomic distribution of P53 and γH2AX in cells expressing α-Synuclein. Genomic distribution of P53 and γH2A peaks in each sample.

Supplementary Figure 3. Changes in the DNA repair genes by α-Synuclein expression. (a) A diagram showing genes involved in the single-strand and double-strand DNA repairs. The genes with red circles are tested for differential expression. (b) PARP1, agene involved in the single strand repair and DSB HR repairs, is tested for expression change with α-synuclein levels. Note that PARP1 decreases, rather than increases, with α-synuclein expression, showing that SSB and DSB HR pathways are downregulated. (c) Rad51, a gene important for DSB HR repair, decreased with α-synuclein expression. (d,e) ERCC1 and XRCC1, genes important for SSB NER and BER respectively, remained the same or decreased with α-synuclein expression.

Supplementary Figure 4. Changes in the γH2AX levels with a longer expression of α-synuclein. The cell number with γH2AX foci and γH2AX foci number per cell increased dramatically with a longer expression of α-synuclein (up to day 10). (scale bar: 20 μm).

Supplementary Figure 5. Real-time PCR analysis of p16, p21, and p53 genes in WT and α-syn Tg mice at 3 months. The real-time PCR results of p16, p21, and p53 genes in each WT and α-syn Tg at 3 mo. of age.

a

DNA repair

Filter: 3	Fold change	p-value	Average of Normalized RC (log2)			
Gene Symbol	dSY-a-4 /dSY-LacZ	dSY-a-4 /dSY-LacZ	dSY-LacZ	dSY-a-4	Description	Gene Type
BTG2	2.883	0.019	11.225	12.753	BTG family, member 2	protein-coding
DDB2	2.447	0.014	11.440	12.731	damage-specific DNA binding protein 2, 48kDa	protein-coding
RPS27L	2.263	0.001	8.539	9.718	ribosomal protein S27-like	protein-coding

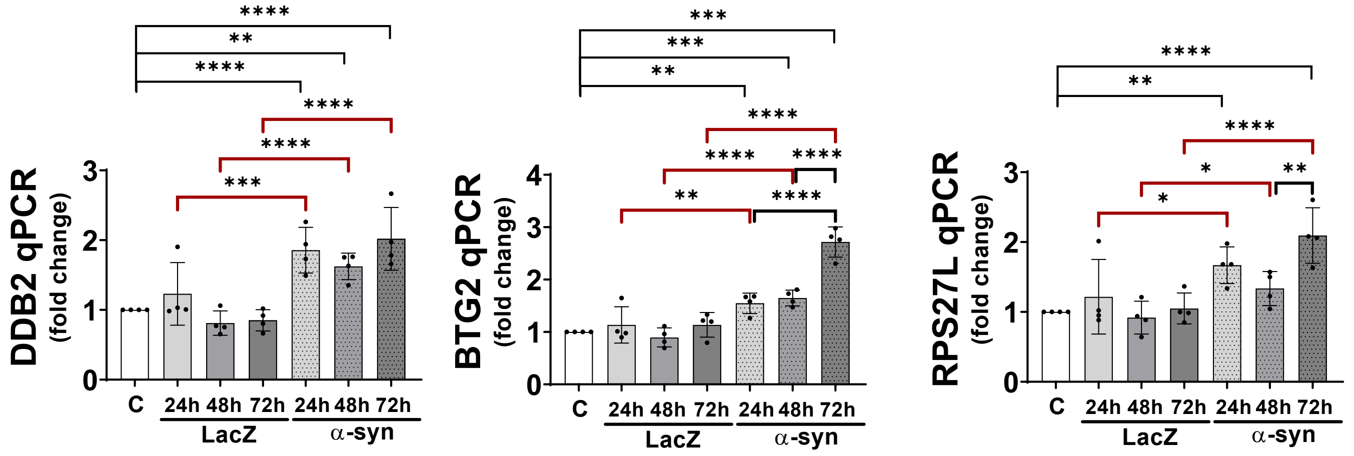

b

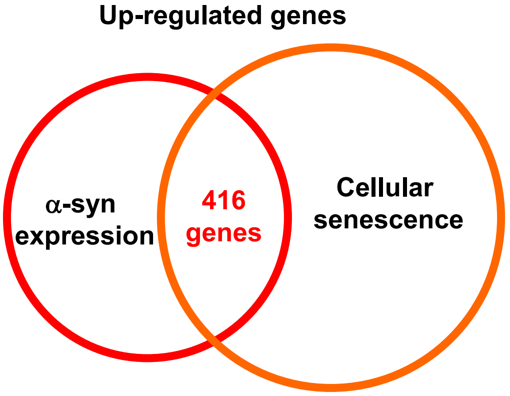

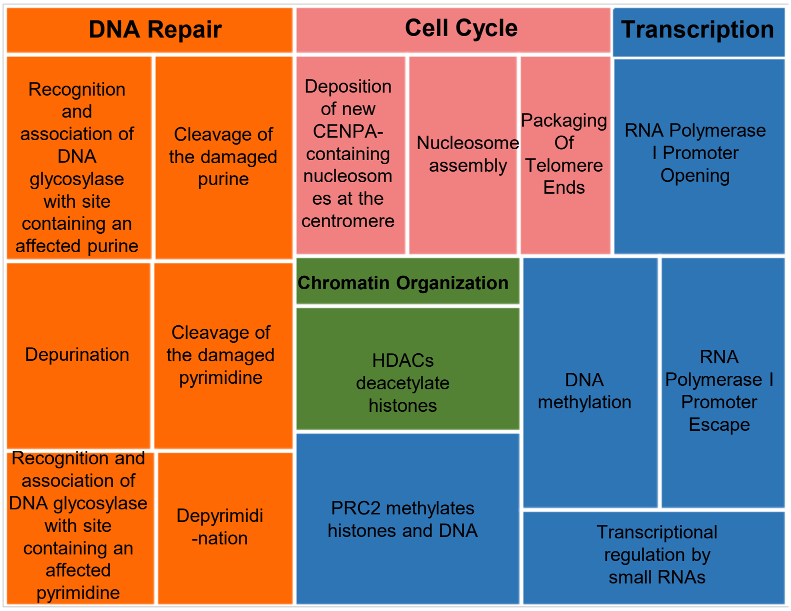

Supplementary Figure 1

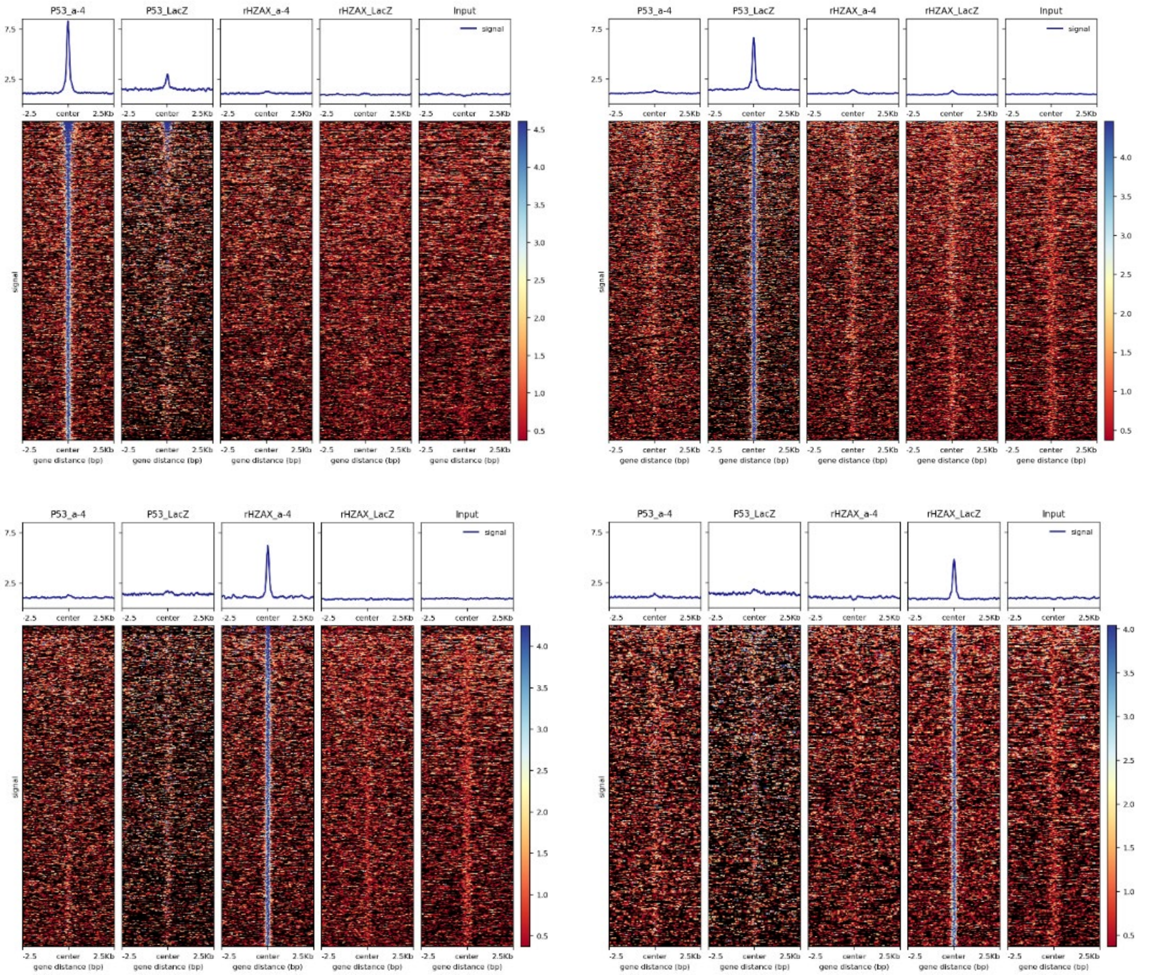

Supplementary Figure 2

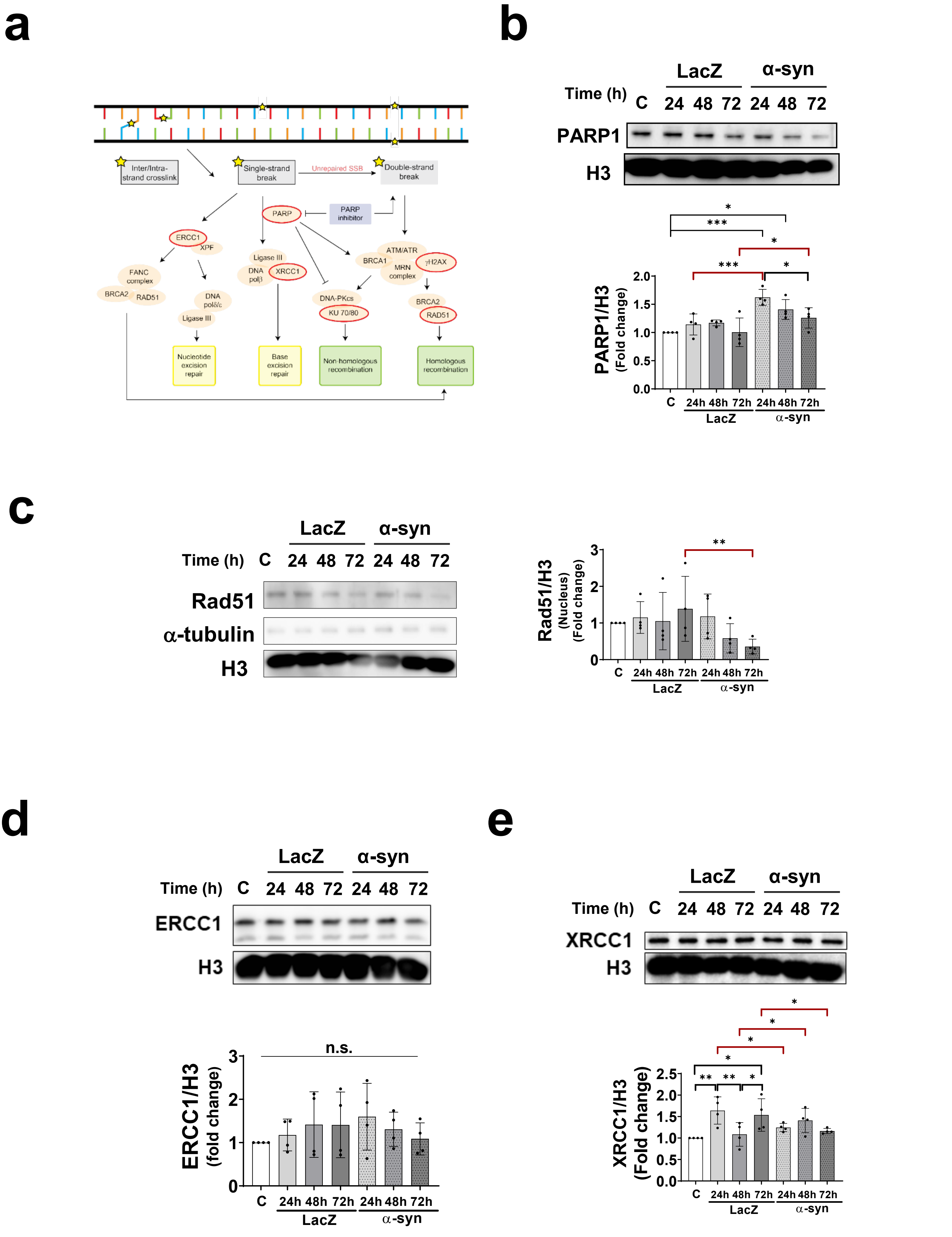

Supplementary Figure 3

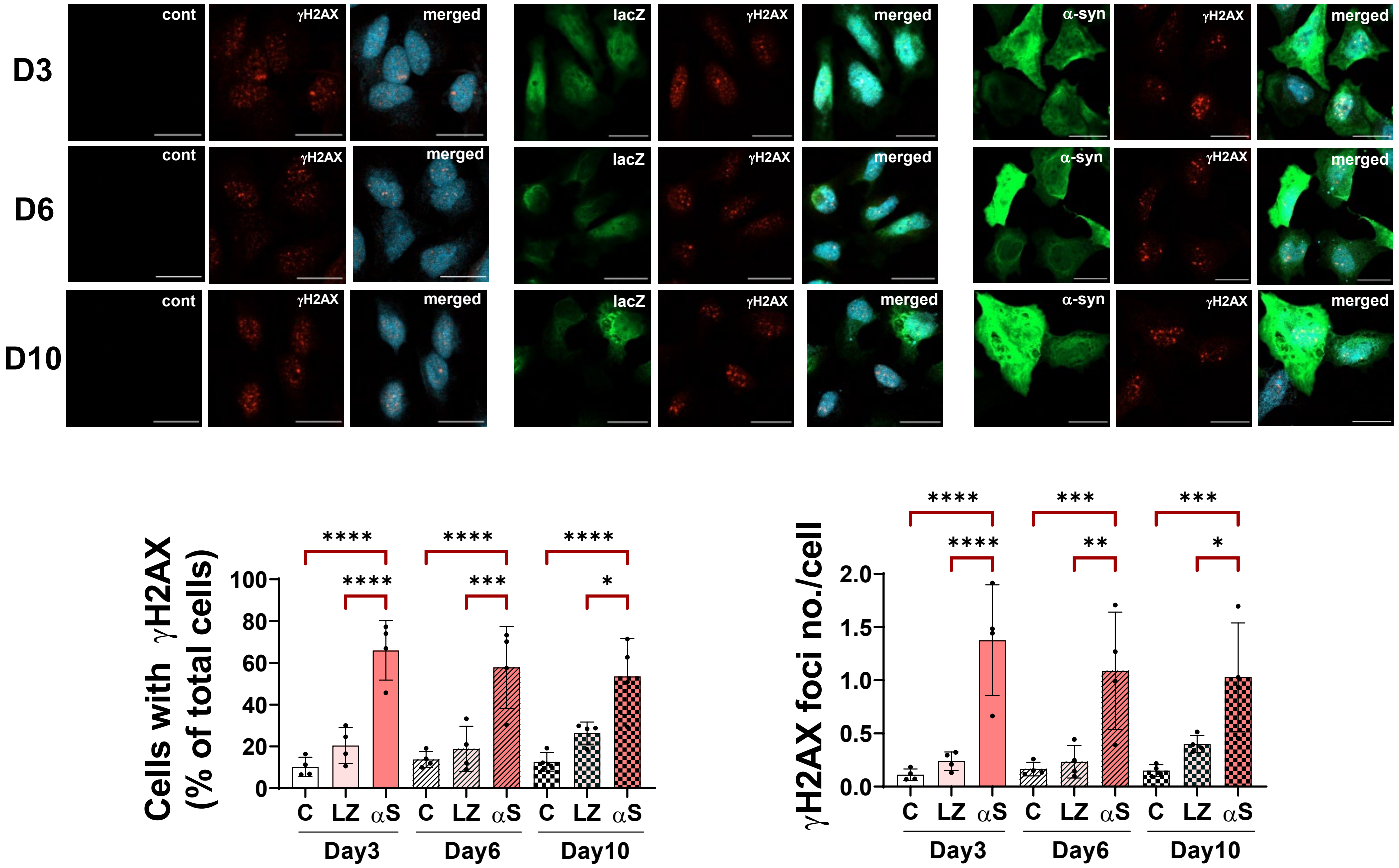

Supplementary Figure 4

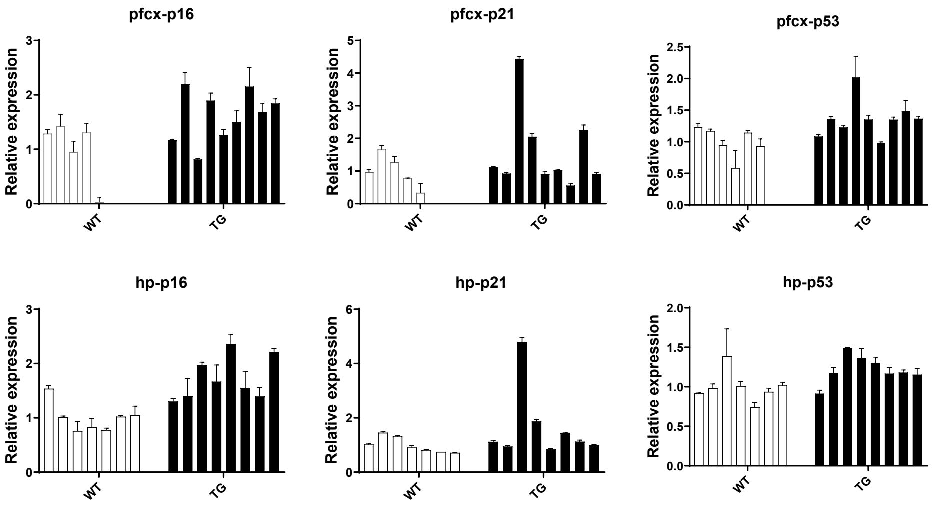

Real time PCR

Supplementary Figure 5
